# Künstliche Intelligenz-unterstützte Behandlung in der Rheumatologie: Grundlagen, aktueller Stand und Ausblick

**DOI:** 10.1007/s00393-021-01096-y

**Published:** 2021-10-07

**Authors:** Thomas Hügle, Maria Kalweit

**Affiliations:** 1 Abteilung Rheumatologie, Universitätsspital Lausanne (CHUV) und Universität Lausanne, Avenue Pierre-Decker 4, 1011 Lausanne, Schweiz; 2 Institut für Informatik, Albert-Ludwigs-Universität Freiburg, Universität Freiburg im Breisgau, Georges-Koehler-Allee 80, 79110 Freiburg im Breisgau, Deutschland

**Keywords:** Entscheidungssysteme, Algorithmen, Automatisierte Bilderkennung, Therapieempfehlungen, Entscheidungsunterstützung, Decision systems, Algorithms, Automated image recognition, Treatment recommendations, Decision support

## Abstract

Computergesteuerte klinische Entscheidungssysteme finden seit Längerem Einzug in die Praxis. Deren primäre Ziele sind die Verbesserung der Behandlungsqualität, Zeitersparnis oder Fehlervermeidung. Meist handelt es sich um regelbasierte Algorithmen, die in elektronische Patientenakten integriert werden, z. B. um Medikamenteninteraktionen zu erkennen. Durch künstliche Intelligenz (KI) können klinische Entscheidungssysteme disruptiv weiterentwickelt werden. Aus Daten wird durch maschinelles Lernen konstant neues Wissen geschaffen, um individuelle Krankheitsverläufe bei Patienten vorherzusagen, Probleme schneller zu erkennen, Phänotypen zu identifizieren oder die Therapieentscheidung zu unterstützen. Solche Algorithmen für rheumatologische Erkrankungen gibt es bereits. Am weitesten fortgeschritten hierbei sind die automatisierte Bilderkennung sowie Vorhersagen zum Krankheitsverlauf bei der rheumatoiden Arthritis. Von nutzerfreundlichen, durch Schnittstellen vernetzten KI-Entscheidungssystemen kann aber noch nicht gesprochen werden. Zudem sind die Algorithmen oft noch nicht genügend validiert und reproduzierbar. Anstatt die KI-unterstützte Wahl der Behandlung dem Arzt oder der Ärztin vorzugeben, wird KI eher als hybride Entscheidungsunterstützung dienen – immer unter Einbezug sowohl des Experten als auch des Patienten. Es gibt zudem ein großes Bedürfnis nach Sicherheit durch nachvollziehbare und auditierbare Algorithmen, um die Qualität und Transparenz von KI-unterstützten Therapieempfehlungen nachhaltig zu gewährleisten.

## Abstract (en)

Computer-guided clinical decision support systems have been finding their way into practice for some time, mostly integrated into electronic medical records. The primary goals are to improve the quality of treatment, save time and avoid errors. These are mostly rule-based algorithms that recognize drug interactions or provide reminder functions. Through artificial intelligence (AI), clinical decision support systems can be disruptively further developed. New knowledge is constantly being created from data through machine learning in order to predict the individual course of a patient’s disease, identify phenotypes or support treatment decisions. Such algorithms already exist for rheumatological diseases. Automated image recognition and disease prediction in rheumatoid arthritis are the most advanced; however, these have not yet been sufficiently tested or integrated into existing decision support systems. Rather than dictating the AI-assisted choice of treatment to the doctor, future clinical decision systems are seen as hybrid decision support, always involving both the expert and the patient. There is also a great need for security through comprehensible and auditable algorithms to sustainably guarantee the quality and transparency of AI-assisted treatment recommendations in the long term.

Der Begriff künstliche Intelligenz (KI) umfasst ein breites Feld an Computeranwendungen, die kognitive Funktionen wie *lernen, planen, erkennen, entscheiden* übernehmen und den Menschen bei bestimmten Aufgaben unterstützen können [1]. Die KI-Forschung existiert seit den 1950er-Jahren und wird stark durch das maschinelle Lernen geprägt. Hierfür werden vorhandene Daten benötigt, die zunächst als Trainingsset (z. B. 80 % der Daten einer Patientenkohorte) eingesetzt werden, um eine vorgegebene Aufgabe zu erlernen. In einem zweiten Schritt wird die Strategie in einem Testset aus den verbleibenden 20 % der Patientenkohorte oder einem zusätzlichen unabhängigen Datensatz validiert. Ein Algorithmus nutzt diese Daten, um als komplexe mathematische Funktion ein Modell als Antwort auf ein klinisches Problem zu erlernen. Dieses Modell wird durch Eingangsvariablen sowie Zielvariablen festgelegt. Stellschrauben des Modells sind sog. Hypervariablen, die dessen Funktionsweise bestimmen und eingrenzen.

In der Medizin sind KI-Anwendungen in jenen Gebieten bereits am meisten verbreitet, in denen große Mengen an Daten zur Verfügung stehen und ein Bedürfnis nach Automatisierung vorliegt [2]. Hier hat sich die Radiologie mit der automatisierten Bilderkennung z. B. zur Diagnose von Tumoren, Frakturen, aber auch rheumatischen Pathologien besonders hervorgetan [3]. Die Entwicklung und Qualität dieser Anwendungen sind in den letzten Jahren derart fortgeschritten, dass zertifizierte KI-Produkte bereits in vielen Radiologiezentren eingeführt werden. KI-unterstützte Prozesse in der Bildgebung unterstützen in erster Linie den Radiologen, greifen aber indirekt auch in den Prozess der Therapieentscheidung ein. Interessanterweise werden KI-Anwendungen von Radiologen – zumindest aus unserer Erfahrung – keinesfalls als Gefahr, sondern als eine moderne digitale Unterstützung wahrgenommen.

Der Begriff Clinical-Decision-Support-Systems (CDSS) beschreibt digitale Anwendungen, die nutzerorientiert dazu dienen, Behandlungsabläufe zu verbessern. Wissensbasierte CDSS gibt es schon seit Längerem, sie beruhen auf bekannten Regeln und generieren durch ihre Algorithmen kein neues Wissen. So kann vor möglichen Medikamenteninteraktionen gewarnt oder an Vorsorgeuntersuchungen oder Impfungen erinnert werden. Dennoch können wissensbasierte CDSS aktiv in den Behandlungsprozess eingreifen, indem z. B. eine Treat-to-Target-Strategie stringenter umgesetzt wird oder unsinnige oder potenziell gefährliche Handlungen verhindert werden.

Bei KI-unterstützten Therapieentscheidungen greifen aus bestehenden Daten gelernte Algorithmen in den Behandlungsprozess ein und erlernen im besten Fall ständig neue, effizientere Lösungen und verringern reines Ausprobieren, was aufgrund fehlender Daten in der Rheumatherapie heute leider noch tagtäglich stattfindet. Der überwiegende Teil der heute eingesetzten KI-Modelle bedient sich des maschinellen Lernens, also Lernprozessen aus dokumentierten früheren Erfahrungen (Abb. 1), die hier im rheumatologischen Kontext erläutert werden sollen.

**Figure 1:**
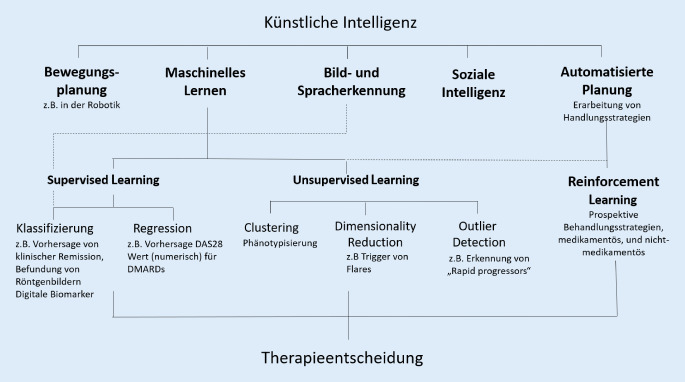

Bei KI-Entscheidungssystemen ging man lange von dem Konzept aus, dass dem Kliniker eine Entscheidung vorgegeben wird. Es stellt sich immer mehr heraus, dass durch den Computer unterstützte Entscheidungen nur durch eine aktive Interaktion mit Kliniker und Patient realisierbar sind [4]. Wie diese Interaktion genau aussieht, soll hier diskutiert werden.

## Algorithmen als Problemlöser

Jeder medizinischen Innovation liegt in der einen oder anderen Form die Lösung eines Problems zugrunde. Klassische statistische Modellrechnungen sind in der Lage, effizient Zusammenhänge zwischen Daten herzustellen oder Wahrscheinlichkeiten für bestimmte Ereignisse in einem Krankheitsverlauf zu errechnen [5]. Sie sind aber weniger gut geeignet, um dem Kliniker konkrete Lösungen anzubieten, und v. a. sind diese Modelle nicht in der Lage, sich den Daten anzupassen oder ihre Leistung selbstständig aufgrund neuer, bislang unbekannter Daten zu verbessern.

Im maschinellen Lernen werden mittels Algorithmen (Handlungsvorschriften) Funktionen approximiert, z. B. durch tiefe neuronale Netze, Entscheidungsbäume oder Kombinationen (= Ensembles) von Entscheidungsbäumen, die im Englischen auch Random Forests genannt werden. Im Deep Learning („tiefes Lernen“), einem Teilbereich des maschinellen Lernens, werden tiefe neuronale Netze eingesetzt, in denen – angelehnt an das menschliche Gehirn – künstliche Neuronen als mathematische Operationen in Schichten angeordnet sind und Signale weiterverarbeiten [6]. Die Anordnung der Operationen resultiert in einer komplexen, nichtlinearen Funktion. Deep Learning wird im Vergleich zu klassischen Methoden des maschinellen Lernens leistungsfähiger, wenn genügend Trainingsdaten vorhanden sind. Die Funktionsweise von tiefen neuronalen Netzen ist allerdings aufgrund ihrer Komplexität schwer zu interpretieren, sie ähneln einer „Black Box“. Für bessere Interpretierbarkeit kann zusätzlich die sog. „feature importance“ (= Einfluss aller Variablen auf die finale Vorhersage/Entscheidung des Modells) berechnet werden.

Im maschinellen Lernen werden mittels Algorithmen Funktionen approximiert um konkrete Aufgaben zu lösen

In jedem Fall muss zuvor die gewünschte Ausgangsvariable als individuelle Antwort auf das klinische Problem festgelegt werden. In den meisten Fällen handelt es sich um eine Klassifizierung in eine zuvor festgelegte Gruppe, z. B. Vorliegen eines Osteophyten auf dem Röntgenbild in die Kategorie ja oder nein. Zuvor muss der Maschine die Gruppe verständlich gemacht werden. Bei der Bilderkennung geschieht dies durch die Segmentierung, bei der Experten auf Probebildern z. B. Osteophyten oder Erosionen markieren. Während in einer Klassifizierung eine „Klasse“ vorhergesagt wird, erlernt ein KI-Model durch Regressionsanalysen die Vorhersage einer kontinuierlichen Variablen. Bezogen auf das Beispiel der Wahl des richtigen Medikamentes, geht es bei Klassifizierung um die Vorhersage, mit welchem Medikament das bessere Ergebnis (z. B. Remission oder nicht) erreicht werden kann. Durch Regression kann eine kontinuierliche Variable, wie der erwartete DAS(Disease Activity Score)28-Wert unter Anwendung eines Medikaments, vorhergesagt werden. Welche die jeweils beste Zielvariable ist, hängt von der Problemstellung ab. Es könnte nach der bestmöglichen DAS28-Reduktion gefragt werden oder dem besten „drug survival“ oder der höchsten Lebensqualität. Die Bestimmung der Zielvariablen und der beeinflussbaren Eingangsvariablen ist ein interaktiver Prozess zwischen Arzt und Patient. KI-basierte Lösungsansätze gibt es bereits als App für Patienten mit autoimmunen Erkrankungen zur Identifikation von Faktoren, die Krankheitsschübe auslösen [7]. Ohne einen Arzt zu konsultieren, können Patienten eine Zielvariable wählen (z. B. Müdigkeit) und eine Auswahl an Variablen wie Physiotherapie, Ernährung, Sport, Medikamente etc. als „patient reported outcomes“ dokumentieren. Danach wird dem Benutzer angezeigt, welche der Variablen den wichtigsten Einfluss auf die Zielvariablen hat bzw. welche Faktoren am ehesten einen Flare auslösen (Clinical study identifier NCT03426384).

Andere Methoden des maschinellen Lernens können neben der Detektion von Triggern, Krankheiten und Vorhersagen der Krankheitsprogression auch mittel- und langfristige Behandlungsansätze ermitteln. Heute wird hierfür v. a. das Reinforcement Learning („bestärkende Lernen“) genutzt, das weiter unten näher erläutert wird und sowohl durch gute als auch negative Erfahrungen lernen kann. Durch die Maschine verursachte negative Entscheidungen (wie bei Schachcomputern z. B. ein Bauernopfer) sind in der Medizin allerdings zu vermeiden, da diese möglicherweise ethisch nicht vertretbar sind. Somit können nicht alle möglichen Strategien vom Algorithmus evaluiert werden und müssen unter strengen Regeln eingeschränkt und überwacht werden. Auf der anderen Seite soll und muss aus unbeabsichtigten menschlichen Fehlern bzw. aus marginalen Fehlern durch die Maschine, selbstverständlich hinzugelernt werden.

## Supervised und Unsupervised Learning

Das Supervised Learning („überwachtes Lernen“) ist die Methode des maschinellen Lernens, die am häufigsten angewendet wird. Sie beruht darauf, dass Funktionen (Zuordnungen von Ein- und gewünschten Ausgaben), wie oben beschrieben, durch „Trainingssets“ anhand vorgegebener Beispiele gelernt werden. Die zu bearbeitende Information wird dem Modell von Ärzten als „gelabelte“ Ausgabebeispiele vorgegeben, wie z. B. der Krankheitsaktivität oder Röntgenbefunde von Patienten zu bestimmten Zeitpunkten. Dann wird die Maschine darauf trainiert, die entsprechenden Labels auch für neue Datenpunkte als Ausgabe zu lernen. Das heißt, das Modell kann z. B. erkennen, ob es sich um einen Patienten handelt, bei dem ein Flare bevorsteht oder nicht (Kategorie Flare ja oder nein). Bei der Bilderkennung verhält es sich ähnlich. Die Aufgabe eines Algorithmus kann z. B. sein, eine Erosion in einem Röntgenbild automatisch zu erkennen. Die Klassifizierung lautet hier Erosion ja/nein. Auch hier müssen die Daten für die Maschine aber erst von Experten gelabelt werden. Dies wird Segmentierung genannt, bei der Teilbereiche der Bilder (z. B. Gelenke) als „region of interest“ markiert werden. Segmentierung kann für den Algorithmus den Lernprozess vereinfachen, indem auf bestimmte Pixel hingewiesen wird, die für das vorgegebene Label „Erosion“ am wichtigsten sind.

Supervised Learning ist die Methode des maschinellen Lernens, die in der Medizin am häufigsten angewendet wird

Beim Unsupervised Learning („unüberwachtes Lernen“) werden Modelle aus nicht gekennzeichneten Daten erstellt. In großen Datenbanken wie einer elektronischen Krankenakte könnte dieses Modell selbstständig bisher nicht bekannte Gemeinsamkeiten bzw. Unterschiede („outlier“) bei Patienten mit einer Krankheit finden. Clusteranalysen helfen dabei, Patienten in bestimmte Gruppen einzuteilen, die sich speziell verhalten und gut oder schlecht auf bestimmte Medikamente ansprechen. Cluster werden hierbei durch Algorithmen, wie z. B. „hierarchical clustering“ oder „*k*-means“ erlernt. Hier gibt es bereits einige Studien im Bereich der rheumatoiden Arthritis (RA), bei der durch solche Analysen verschiedene Cluster identifiziert wurden. Zum Beispiel war das Scheitern einer Biologikatherapie in einem Cluster mit männlichen Rauchern besonders hoch [8].

## Reinforcement Learning

Das Reinforcement Learning gilt als Königsdisziplin des maschinellen Lernens [9]. Hier lernt der Computer, welche Handlung in bestimmten Situationen langfristig den größten Erfolg mit sich bringt, beispielsweise hinsichtlich einer „anhaltenden Remission“. Hierzu wird zu jeder Aktion ein Feedback einbezogen, das aufzeigt, ob eine Aktion zur aktuellen Zeit gut oder schlecht war, und hilft, eine Belohnungsfunktion zu erstellen. Wie bei der praktischen Rheumabehandlung heute der Fall, spielt hier „trial and error“ eine Rolle – es kann auch durch Negativbeispiele gelernt werden.

Die klinischen Erfahrungen, die Rheumatologen in ihrer Laufbahn mit verschiedenen Biologika gemacht haben, könnte ein Algorithmus mithilfe einer großen Datenbank um ein Vielfaches übersteigen. Die „Kunst“ der richtigen Therapieentscheidung wird beim Reinforcement Learning durch eine komplexe mathematische Funktion in Form eines Regressionsmodells wie einem neuronalen Netz getätigt. Allerdings kann es hier durch die Belohnungsfunktion trotz Regeln zu unerwarteten Ereignissen kommen. Klinische Studien mittels Reinforcement Learning im Bereich der Rheumatologie wurden unseres Wissens bislang noch nicht durchgeführt. Eine Studie aus einem anderen Feld, die Aufmerksamkeit erregt hat, war die Steuerung von Flüssigkeit und Katecholaminen durch Reinforcement Learning auf der Intensivstation [10]. Das Modell war der menschlichen Entscheidung überlegen, die besten Ergebnisse wurden jedoch erzielt, wenn Mensch und Maschine zur gleichen Entscheidung kamen.

## Klassisches maschinelles Lernen versus Deep Learning

Das maschinelle Lernen ist keine neue Erfindung, sondern geht, wie oben beschrieben, auf die 1950er-Jahre mit ihren klassischen Methoden wie „k-Nearest-neighbors“, „support vector machines“ oder Entscheidungsbäumen zurück. Schon zu diesem Zeitpunkt wurden künstliche neuronale Netze entwickelt, die an der Funktionsweise der Neuronen im menschlichen Gehirn angelehnt und in Schichten aufgebaut sind. Allerdings wurde das Deep Learning erst in den letzten Jahren erfolgreich, als diese Netze „tief“ genug geworden sind und durch starke Prozessoren und Grafikkarten genügend Rechenleistung zur Verfügung stand. Neuronale Netze sind im Falle eines genügend großen Datensets mächtiger als klassische Methoden des maschinellen Lernens und können besser auf komplexen klinischen Daten lernen und generalisieren. Es gibt verschiedene Architekturen von tiefen Netzen, wie z. B. „fully-connected neural networks“, „convolutional neural networks“ oder „recurrent neural networks“, oder auch Architekturen, speziell angepasst für klinische Daten, die anhand der Art der Eingabevariablen (numerische Daten, Bilder und Zeitreihen) ausgewählt werden [11]. Wichtig zu verstehen ist, dass neuronale Netze als Approximation von Funktionen sowohl für Supervised oder Unsupervised Learning sowie für Reinforcement Learning eingesetzt werden können.

## Automatisierte klinische Entscheidungssysteme in der Rheumatologie

**Figure 2:**
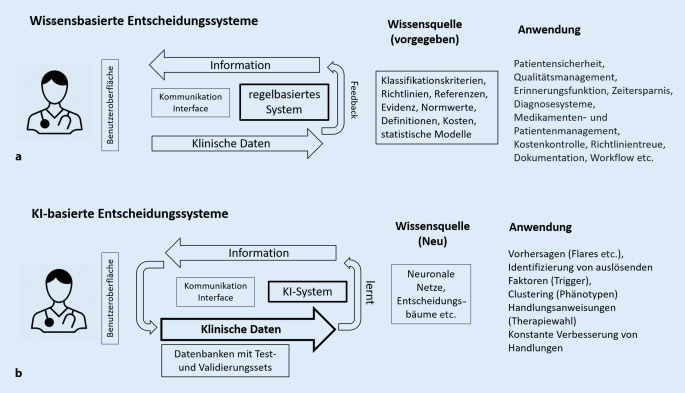

Digitale klinische Entscheidungssysteme („clinical decision support systems“, kurz CDSS) wurden geschaffen, um datengesteuert die Behandlungsqualität zu verbessern und durch Automatisierung den Kliniker zu unterstützen. Meist erfolgt dies durch Entscheidungsbäume, in denen vorgegeben ist, was in welcher Situation zu tun ist. Dies kann eine regelbasierte Assistenz sein z. B. durch Erinnerungen und Warnungen (z. B. bei Medikamenteninteraktionen) und geht weiter zur automatischen Überprüfung der Diagnose bis hin zur Einhaltung von Therapierichtlinien und Treat-to-Target-Strategie (Abb. 2). CDSS wurden bereits vor längerer Zeit in der Rheumatologie eingeführt, teilweise noch papierbasiert [12]. In der elektronischen Krankenakte kann zudem durch ein CDSS bei RA-Patienten angezeigt werden, ob durch Risikofaktoren wie Geschlecht, CRP (C-reaktives Protein), Anti-CCP (zyklische citrullinierte Peptide) oder Erosionen im Röntgenbericht ein hohes Risiko für eine Progression vorliegt. Durch das „Natural Language Processing“ können KI-basiert Informationen mittlerweile auch automatisch aus Berichten erkannt und in Kontext gesetzt werden z. B. zum Finden von Patienten mit Spondylarthritis aus elektronischen Krankenakten [13]. In einer spanischen Studie wurde ein Patient Decision Aid (PDA) bei Patienten mit moderater bis schwieriger RA untersucht. Die Funktionsweise der Applikation liegt primär in der gezielten Information für Patienten, durch die Konflikte bei der Behandlung reduziert werden konnten [14].

Durch mobile Apps können zudem Daten im Bereich Medikamentenmanagement (z. B. Compliance) und „patient reported outcomes“ (PROs) über eine Schnittstelle in ein CDSS integriert werden und somit dem Rheumatologen Entscheidungen zusätzlich erleichtern bzw. Zeit einsparen. Bei der juvenilen Arthritis wurde beim Einsatz eines CDSS eine signifikant verminderte Krankheitsaktivität festgestellt [15]. In der Studie wurde ein klassisches CDSS eingesetzt, also primär eine Unterstützung des Patienten- und Medikamentenmanagements. Man kann bei diesen Funktionen dabei aber nicht von einer Therapieempfehlung und im strengeren Sinne auch nicht von einer personalisierten Behandlung sprechen, da allgemeingültige Regeln angewendet werden.

## Künstliche Intelligenz-unterstützte Therapieentscheidungen

KI-gesteuerte CDSS eröffnen mehr Möglichkeiten und können theoretisch Therapieentscheidungen breiter und flexibler unterstützen als regelbasierte CDSS. Bislang fließen in solche Systeme in erster Linie klinische Daten ein, z. B. aus Registern oder elektronischen Patientenakten. Norgeot et al. konnten Remissionen bei RA-Patienten mithilfe von Daten aus unterschiedlichen amerikanischen elektronischen Krankenakten mit einer Genauigkeit von ca. 90 % voraussagen. Allerdings werden solche Vorhersagen durch lange, stabile Krankheitsverläufe verfälscht, die besonders leicht vorhersagbar sind [16]. In einer kleineren Studie konnten individuelle RA-Flares beim Ausschleichen der biologischen DMARD(„disease-modifying anti-rheumatic drug“)-Therapie durch maschinelles Lernen mit einer Genauigkeit von 80 % vorhergesagt werden. Die wichtigsten Variablen hierfür waren die zugrunde liegende Dosisreduktion, Krankheitsaktivität, Krankheitsdauer und Entzündungsparameter [17]. In einigen RA-Studien wurden neben klinischen Daten auch histologische und molekulare Daten genutzt, um RA-Phänotypen zu identifizieren [18]. Auch das Therapieansprechen von TNF(Tumor-Nekrose-Faktor)-Blockern mithilfe der DNA(Desoxyribonukleinsäure)-Methylierung [19] oder genetischen Daten [20] wurde untersucht. Bei der Behandlung mit TNF-Blockern wurde hier eine korrekte Vorhersage zum Ansprechen bzw. Nichtansprechen in 80–90 % der Fälle erreicht. Die Zusammenführung von klinischen (z. B. aus elektronischen Patientenakten) und biomedizinischen Daten ist hierbei nicht trivial und bedarf spezieller Netzwerke [21]. Ähnliches gilt für Daten von Wearables, mit denen durch maschinelles Lernen zuverlässig Flares detektiert werden konnten [22].

Während bei wissensbasierten CDSS eine generelle Handlungsanweisung ausgegeben wird, z. B. für eine Treat-to-Target-Strategie, können KI-Modelle flexibler vorgehen und Vorhersagen machen, welches Medikament in einer bestimmten Situation am ehesten das „Target“ erreicht (Abb. 2b). KI-gesteuerte verbesserte Ergebnisse werden wiederum in der Datenbank erfasst und fließen je nach KI-Modell nach Updates oder auch direkt in die Handlungsanweisungen ein.

In einem aktuellen Review über maschinelles Lernen bei Autoimmunerkrankungen wurde die Literatur in folgende Themen eingeteilt: Patientenidentifikation, Risikovorhersage, Diagnose, Phänotypisierung sowie Vorhersagen zur Krankheitsprogression, Outcome oder Management [23]. Bezüglich Therapieentscheidung erscheinen hier Phänotypisierung und Vorhersage des Therapieverlaufs am relevantesten, daher soll auf diese hier weiter eingegangen werden.

Durch Supervised Learning kann der Computer den Kliniker und Patienten vor anstehenden Krankheitsschüben warnen, auch wenn klinisch noch keine Gelenkschwellung oder CRP-Erhöhung vorliegt. Anstatt zu reagieren, kann die Behandlung also schon früher, vielleicht sogar prophylaktisch, umgestellt werden, was möglicherweise zu geringeren Medikationsdosen führt, weniger Nebenwirkungen mit sich bringt und Kosten sparen könnte.

Bereits 2016 erreichte ein neuronales Netzwerk zur Vorhersage auf das Ansprechen auf Infliximab eine Accuracy von 92 % mit nur 9 klinischen Variablen [24]. Auch die Entscheidung zur Infliximab-Dosiseskalation konnte durch maschinelles Lernen effizient klassifiziert und damit vorhergesagt werden [25]. Maschinelles Lernen konnte das Ansprechen auf eine Tocilizumab-Monotherapie vorhersagen, und zwar ähnlich gut aus einem Real-world-Datensatz wie aus verschiedenen kontrollierten Studien [26].

Durch Regressionsanalysen kann die Krankheitsaktivität auch numerisch z. B. durch den DAS28-CRP unter bestimmten Bedingungen (Medikament A, B oder C) vorhergesagt werden. In diesem Szenario kann ein CDSS nicht nur die Adhärenz einer Treat-to-Target-Strategie überprüfen, sondern eine möglicherweise effizientere Treat-to-predicted-Target-Strategie ermöglichen. Hypothetisch verändert sich durch verbesserte KI-gesteuerte Therapieentscheide auch die Datenbank an sich und führt dann wiederum zu verbesserten Voraussagen.

Durch Clusteranalysen („Clustering“) von EPAs (elektronische Patientenakten) oder Registern können neue Rheumaphänotypen identifiziert werden, wodurch sich die zukünftige „patient journey“ leichter einordnen lässt. Momentan ordnen wir ein Overlap-Syndrom primär nach Klinik und Autoantikörpermuster ein. Clustering erlaubt die Einordnung auf einem höheren Niveau inklusive Outcome und Ansprechen auf Medikamente. Wir postulieren, dass Clustering in Zukunft expertenbasierte Klassifikationskriterien verbessern wird. Dies erfolgt meist durch Unsupervised Learning, das auch bei einer RA-Studie über Pathotypen und Genexpression eingesetzt wurde und 3 verschiedene Arten von Synovitis ermittelt hat [18].

Reinforcement Learning erlaubt auf Basis von konstanter Rückmeldung von Behandlungsergebnissen (z. B. via App), Therapien immer weiter zu verbessern, um auch mittel- und langfristig die besten klinischen Resultate zu erreichen.

Die ständige Verbesserung durch Rückmeldung ist nicht auf KI-Methoden beschränkt, sondern kann auch klassisch oder stochastisch erfolgen. Möglicherweise kommen in Zukunft auch Kombinationen aus maschinellem Lernen und klassischen Modellen zur Anwendung.

## Rolle von Arzt und Patient

Maschinelles Lernen ist anfällig für unvollständige, falsche und unspezifische Daten (auch „garbage in garbage out“ genannt). Der Arzt muss dem Lernsystem zunächst deshalb vorgeben, welche Informationen für eine Therapieentscheidung in Betracht gezogen werden müssen.

Andererseits ist es möglich, Patienten die Wahl zu lassen, welches Outcome für sie am wichtigsten ist. Dies kann anstatt eines Markers für Krankheitsaktivität auch ein Symptom wie Müdigkeit sein. Dann wird die KI spezifisch hierfür trainiert, um die effizienteste medikamentöse oder nichtmedikamentöse Intervention zu empfehlen.

**Figure 3:**
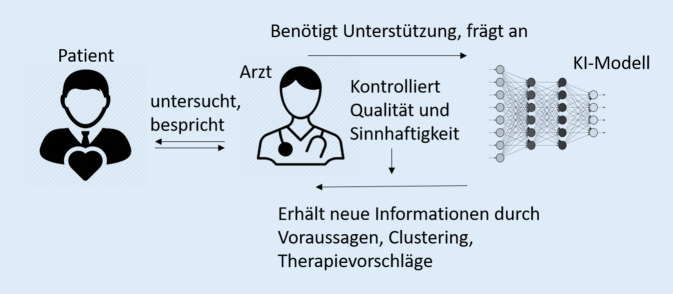

Schließlich bleiben Therapieentscheidungen ein heterogener Abwägungsprozess, bei dem Kliniker immer verschiedene Arten von Informationen in Betracht ziehen, um ein kohärentes und adäquates Bild der Patientensituation zu erhalten und eine verantwortungsvolle und gerechtfertigte Schlussfolgerung zu ziehen. Hierfür trägt der Arzt eine sog. erkenntnistheoretische Verantwortung („epistemological responsibility“). Beim Einsatz von KI in der Klinik spricht man deshalb von „Hybrid-Intelligenz“ aus Maschine und Experte (Abb. 3). Der Arzt muss dabei immer die Plausibilität der vorgeschlagenen Entscheidung überprüfen und wird deshalb voraussichtlich die Verantwortung und Haftung der Behandlung übernehmen.

Aufgrund der potenziellen Auswirkungen von KI-unterstützten CDSS werden Richtlinien zu deren Entwicklung und Anwendungen erstellt und ständig weiterentwickelt [27].

Parallel zu CDSS werden zunehmend komplett digitale Lösungen in Form von Apps entwickelt. Patienten können als „quantified self“ individuelle Daten eingeben sowie Trends und Therapieziele selbstständig überwachen. Das Erkennen von Triggern von autoimmunen Erkrankungen durch maschinelles Lernen wird bereits jetzt als Applikation angeboten (s. mymee.com). Hier werden v. a. Informationen über Bewegung, Ernährung, Lifestyle etc. gesammelt, und deren Bedeutung für einzelne Symptome wird analysiert. Neben Verhaltensänderungen sollen Apps auch „Drug like“-Effekte erzielen, wie dies für psychiatrische Erkrankungen – aber noch nicht für die Rheumatologie – bereits gezeigt wurde [28].

## Keine Black Box – interpretierbare, auditierbare und „ethische“ künstliche Intelligenz

Während sich Entscheidungen bei regelbasierten Algorithmen nachvollziehen lassen, ähneln manche Modelle des maschinellen Lernens mehr einer „Black Box“. Dies kann zu ethischen Problemen bei der Anwendung von KI führen [29]. Mittlerweile wurden deshalb von offizieller Seite Mindestanforderungen zur Transparenz von Algorithmen publiziert, wie die „Barcelona declaration for the proper development and usage of artificial intelligence in Europe“; [30]. Der Begriff „erklärbare KI“ breitet sich aus. Auf einer höheren Ebene wird auch bereits diskutiert, inwieweit das sog. maschinelle Verhalten von Algorithmen definiert sein muss. So kann definiert werden, wie aggressiv ein Algorithmus bei der Therapieumstellung ist und z. B. bereits auf kleine DAS28-CRP-Schwankungen reagiert. In Zukunft muss festgelegt sein, welches langfristige Ziel ein Algorithmus verfolgt und ob die Kosteneffektivität einer Behandlung im Algorithmus eine Rolle spielen darf. Nimmt man beispielsweise „drug survival“ als Ergebnis, wird der Algorithmus alles tun, damit der Patient möglichst lange ohne Therapiewechsel bleibt. Auf der anderen Seite können neue Medikamente in Algorithmen Nachteile haben, da weniger Daten existieren. Ein weiteres Risiko beim Einsatz von Algorithmen ist eine Monopolisierung, d. h. das beste System wird durch immer größere Nutzung immer besser und damit nicht einholbar. Es stellt sich auch die Frage, wem zukünftig Algorithmen gehören bzw. wer sie nutzen darf.

In Zukunft muss festgelegt sein, welches langfristige Ziel ein Algorithmus verfolgt

Außerdem muss dem Benutzer angezeigt werden ob die klinischen Daten, die ein KI-System trainieren, bestimmte Qualitätsansprüche erfüllen. Ansonsten sollte von der Maschine kein Therapievorschlag abgegeben werden. Für Nicht-Informatiker ist es bereits sehr hilfreich, die Begriffe zu kennen, mit denen Algorithmen bzw. KI-Systeme charakterisiert werden (Tab. 1). Als längerfristiges Ziel sollten medizinische KI-Systeme standardisiert miteinander verglichen werden, um deren Qualität besser einschätzen zu können.

**Table 1:** 

Wert	Definition
Accuracy	Messung der Leistung eines KI-Modells. Proportion der korrekt vorhergesagten Datenpunkte aus allen Datenpunkten
Area under the Curve (AUC), ROC (Receiver Operating Characteristics), AUC-ROC	Wichtige Leistungsmessung eines Algorithmus bei Klassifizierung
Robustness	Eigenschaft, die charakterisiert, wie robust ein Algorithmus dieselben Ergebnisse bei mehrfachen Trainingsdurchläufen liefert, idealerweise auch auf unabhängigen Datensätzen
Outcome	Meist ist die klinische Zielvariable gemeint, also welcher Wert vorausgesagt werden soll
Accountability	Rechenschaftspflicht. Entscheidungsalgorithmen müssen für den Nutzer ableitbar und erklärbar sein
Constraints	Einschränkungen, die einem Algorithmus vorgegeben werden. Zum Beispiel darf ein Algorithmus keine sinnlosen oder sogar schädlichen medizinischen Handlungsanweisungen geben
Explainability	Erklärbarkeit eines KI-Systems z. B. durch die Sammlung von Merkmalen, die zu einer Entscheidung beigetragen haben
Machine behaviour	Charakter eines KI-Agenten (Steuerelement des Algorithmus) in Bezug auf Sinn und Zweck des Algorithmus sowie dessen soziotechnologische Auswirkungen
Hyperparameter	Abstimmbare Parameter, die vor Beginn des Lernprozesses festgelegt werden, z. B. Lernrate, Momentum, Anzahl von Clustern, Epochen (Durchläufe), Anzahl von Verzweigungen in einem Entscheidungsbaum
Testset	Datensatz von Beispielen zur Auswertung
Validierungsset	Der Validierungsdatensatz bietet eine unvoreingenommene Bewertung der Anpassung eines Modells an den Trainingsdatensatz, während die Hyperparameter des Modells abgestimmt werden

*KI* künstliche Intelligenz

## Implementierung von künstlichen Intelligenz-Systemen in der klinischen Praxis

Die technische Machbarkeit von KI-basierten Therapieentscheidungen wurde oben dargelegt, zumindest unter der Bedingung, dass eine ausreichende und qualitativ genügende Datenmenge vorhanden ist. Die Anwendung dieser Ergebnisse in der klinischen Praxis stellt wahrscheinlich die größere Aufgabe dar. Es bedarf jeder Menge Veränderungsprozesse, um sowohl Kliniker und Patienten an diese neue Art des Handelns zu gewöhnen. Dies gilt umso mehr, da es sich bei jeder Therapieentscheidung letztendlich um eine Abwägung handelt. Hierbei kommen neben der KI auch zwingend das Wissen des Experten und die Entscheidung der Patienten zum Tragen, bei Letzterem auch unter Einbezug von persönlichen, familiären und nichtobjektiven Faktoren. Eine weitere Frage stellt sich, wie Therapieentscheidungen in den zukünftigen klinischen Workflow passen. Für neuere Medikamente stehen weniger Datenpunkte zur Verfügung, somit werden diese von KI-Systemen zunächst sehr wahrscheinlich weniger berücksichtigt. Theoretisch könnten sich KI-Systeme in diesem Zusammenhang sogar als Innovationsbremse herausstellen.

**Figure 4:**
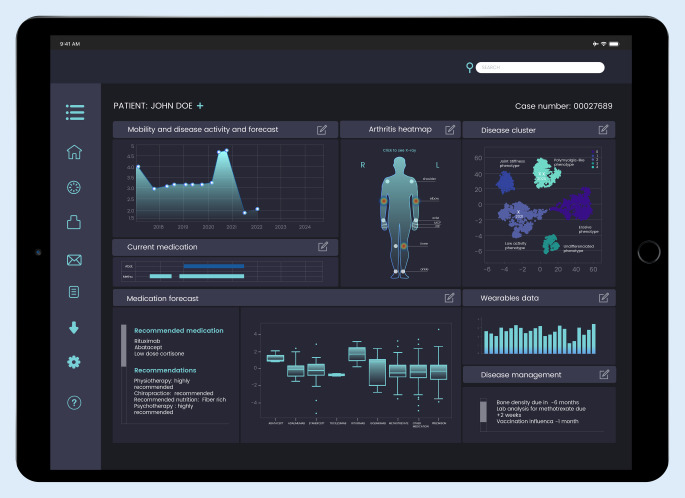

Eine wichtige Frage ist, wie der Anwender in Zukunft mit einem KI-System interagiert. Der Kliniker könnte Informationen als Tabelle erhalten oder als direkte Audio-Antwort über Spracherkennung noch während der Sprechstunde. Um von einem Entscheidungssystem sprechen zu können, wäre aus unserer Sicht eine visuelle Plattform in Form eines Dashboards besser geeignet, das dem Experten verschiedene maschinell gelernte Ergebnisse genauso wie deren Genauigkeit und Unsicherheit anzeigt. Ein visuelles Beispiel eines fiktiven KI-Dashboards ist unter Abb. 4 zu sehen. Zu guter Letzt müssen Sicherheitsmaßnahmen getroffen werden, damit Empfehlungen der KI den Patienten nicht in Gefahr bringen (z. B. Allergie, Niereninsuffizienz etc.). Das hieße, dass bei unvollständigen oder qualitativ schlechten Daten mitunter keine Empfehlung angezeigt wird. Diesbezüglich stellt sich die Frage, wer für die Konsequenzen von KI-Therapieentscheidungen haftet. Es ist davon auszugehen, dass der behandelnde Arzt oder die Ärztin auf alle Fälle die Plausibilität der Empfehlungen prüfen muss und damit verantwortlich ist.

## Fazit für die Praxis

- Aktuelle KI(künstliche Intelligenz)-basierende Ansätze und Algorithmen zur Unterstützung der Rheumabehandlung zielen auf die Vorhersagen von Therapieansprechen oder auf die Identifizierung von Phänotypen ab.
- Algorithmen zur Unterstützung der Therapie werden in Zukunft strengen Sicherheitsanforderungen und einer dauerhaften Evaluation unterliegen müssen.
- Die Auswahl von klinischen Variablen bzw. der Zielvariablen als primär zu behandelndem Symptom oder Aktivitätsscore wird idealerweise von Arzt und Patient aktiv zusammengestellt.
- Als Schnittstellen zwischen KI und Behandler werden wahrscheinlich eigene Plattformen oder integrierte Lösungen in bestehende Oberflächen von Krankenhaus- oder Praxissystemen dienen.
- Patienten werden in Zukunft über Apps ihre Behandlung mitgestalten, indem sie z. B. Trigger für Krankheitsschübe über KI-Lösungen selbst identifizieren oder bei anstehenden Schüben die klinische Kontrolle automatisch vorverlegen.
